# Open Access: The Effect of Neurorehabilitation on Multiple Sclerosis—Unlocking the Resting-State fMRI Data

**DOI:** 10.3389/fnins.2021.662784

**Published:** 2021-05-28

**Authors:** Barbora Bučková, Jakub Kopal, Kamila Řasová, Jaroslav Tintěra, Jaroslav Hlinka

**Affiliations:** ^1^Department of Cybernetics, Faculty of Electrical Engineering, Czech Technical University in Prague, Prague, Czechia; ^2^Department of Complex Systems, Institute of Computer Science of the Czech Academy of Sciences, Prague, Czechia; ^3^Department of Applied Neuroscience and Neuroimaging, National Institute of Mental Health, Klecany, Czechia; ^4^Department of Computing and Control Engineering, University of Chemistry and Technology, Prague, Czechia; ^5^Third Faculty of Medicine, Charles University, Prague, Czechia; ^6^Radiodiagnostic and Interventional Radiology Department, Institute for Clinical and Experimental Medicine, Prague, Czechia

**Keywords:** fMRI, multiple sclerosis, open access, data report, neurorehabilitation

## 1. Introduction

Multiple Sclerosis (MS) is a severe neurological condition, the incidence of which is growing on a global scale, reporting over 2.2 million cases worldwide in 2016 (Wallin et al., 2019). The symptoms associated with the disease vary and include gait and postural control changes, problems with vision, sensory symptoms, cognitive decline, depression, and others (Kister et al., 2013). Despite the seriousness of the condition, its' relative rareness and heterogeneity obstructs the neuroimaging research that could identify brain-dependent patterns that play a role in the disease's progression.

It has been shown that acute relapse or chronic insult in people with MS may stimulate brain plasticity mechanisms—the intrinsic cascades of the brain to functionally and structurally reorganize itself in response to external stimuli. However, a gradual failure of these processes to manage the increasing structural and functional changes caused by demyelination may eventually lead to impairment (Pascual-Leone et al., 2005; Prosperini et al., 2015). It has been hypothesized that by supporting the brain plasticity mechanisms via regular cognitive and motoric exercise, the progression of the disease might be slowed down. The positive effect of neurorehabilitation on brain activity has already been studied to some degree. Rasova et al. reported an increased correlation of fMRI activity among hemispheres following 2 months of eclectic rehabilitative therapy. Furthermore, they also found a decrease of effective connectivity at supplementary motor areas (Rasova et al., 2005, 2015). This evidence was later supported by other groups finding a beneficial functional reorganization in the sensory-motor network after motor rehabilitation (Tavazzi et al., 2018; Fling et al., 2019; Prochazkova et al., 2020). Tomassini et al. (2012) studied brain plasticity for visuomotor practice in MS patients and found it preserved despite the extent of cerebral pathology. Another study observed a correlation between motor improvements and fMRI modifications in MS patients after watching and imitating videos of daily life (Rocca et al., 2019). However, as pointed out in a recent review, most of the research in this domain suffers from the use of small sets of volunteers, which usually does not exceed twenty (Prosperini and Di Filippo, 2019). This raises concerns about the interpretation and generalization of reported findings, and makes the idea of sharing data more desirable (and eventually inevitable) to gain credibility.

Considering the above, we share resting-state fMRI activity profiles across 60 people with MS before and after 2 months of neuroproprioceptive “facilitation and inhibition” treatment. Our goal in sharing the data is to promote further research in this area and improve reproducibility of any future reports. The data were gathered using fMRI as it is a well-established tool in studying neurological conditions (Lee et al., 2013). Its value lies in recording spontaneous low-frequency fluctuations in the blood oxygen level-dependent signal, allowing for the inspection of functional processes across the brain. By measuring the level of temporal dependence (e.g., correlation) of neuronal activity among brain regions, we obtain functional connectivity (FC) (Friston et al., 1993; Lee et al., 2013). FC may be studied at various levels, ranging from the analysis of strengths of correlation between two voxels to the identification and characterization of large-scale networks (Du et al., 2018). The most common approaches include model-driven analyses of connectivity across pre-defined brain regions, and also data-driven approaches based on decomposition methods. Moreover, having two distinct recordings per subject, intra- as well as inter-individual differences may be analyzed. Thus, the dataset could be used to study effects of neurorehabilitation or to reproduce the research investigating resting-state connectivity in MS patients. In the latter case, the data are particularly well-suited for examining interindividual variability in the clinical symptoms. In general, the in-depth investigation of fMRI signals has the potential to shed light on the neural correlates associated with the disease (Enzinger et al., 2016; De Giglio et al., 2018; Péran et al., 2020).

## 2. Methods

### 2.1. Patients

People with MS were recruited from specialized centers in the Czech Republic for 5 years. The recruitment period was spread over two subsequent projects; the pilot project (NCT04448444) ran within the years 2013–2014, with the consecutive project following close in 2015–2017 (NCT04355663). The pre-defined inclusion criteria in both projects consisted of a positive diagnosis of MS (according to the Polman et al., 2011 criteria), Expanded Disability Status Scale (EDSS) of maximum 7.5, neurologically confirmed stable clinical status for a minimum of 3 months prior to the study, notable spastic paraparesis among the symptoms, and the ability to reach a rehabilitation center regularly. The condition for the presence of motor impairment and the closeness of the rehabilitation unit relates to the original project purpose, which studied the effect of motor rehabilitation on clinical as well as neurological biomarkers (see, e.g., Prochazkova et al., 2020). The exclusion criteria were defined as follows: mobility disturbed for reasons other than those related to the disease (fractures, pregnancy, and others), or the presence of other orthopedic, cardiovascular, or neurological conditions. Patients were involved in the study independent of MS phenotype—relapsing-remitting, primary progressive, and secondary progressive.

The presented dataset contains resting fMRI from 60 patients before and after 2 months of ambulatory “facilitation, inhibition” physical therapy (Prochazkova et al., 2020). Participants were assigned to one of three specific variants of the treatment: Motor Program Activating Therapy, Vojta's reflex locomotion, and Functional Electric Stimulation in Posturally Corrected Position. All treatments are based on similar principles. The techniques focus on the appropriate combination of afferent stimuli in pre-defined postural positions activating the motor programs that lead to a motor reaction of the entire body with the following effects:

- muscle synchronization—the co-contraction of an agonist and antagonist
- functional centration—the best possible distribution of the load at the articular surfaces
- the postural stabilization in the sagittal plane across the entire body.

The data description is in Table 1. All participants were informed about the experimental setup and provided written informed consent following the Declaration of Helsinki. The Ethics Committee of the Faculty Hospital Královské Vinohrady approved the design of the studies.

**Table 1:** Data description.

	**Females**	**Males**	**All**
No.	37	23	60
Age	48.0 (22, 70)	44.0 (29, 68)	46.0 (22, 70)
EDSS	4.0 (1, 6)	4.0 (1, 7)	4.0 (1, 7)
MS type RR/SP/PP	21/15/1	13/5/5	34/20/6
Years since diagnosis	13 (1, 38)	11 (1, 22)	12 (1, 38)
Therapy variant FES/MPAT/VRL	6/24/7	7/10/6	13/34/13
BMI	22.7 (16, 35)	23.1 (19, 36)	22.7 (16, 36)

*No., number of patients; EDSS, Expanded Disability Status Scale; RR, relapsing-remitting; PP, primary progressive; SP, secondary progressive; FES, functional electric stimulation; MPAT, motor program activating therapy; VRL, Vojta reflex locomotion; age, EDSS, years since diagnosis, and BMI are listed as median (min, max)*.

### 2.2. Data Acquisition

Imaging was performed using a Siemens Trio 3T equipped with a 12-channel phased-array head coil. The fMRI protocol in the two projects slightly differed. Both acquisitions are described below: **Pilot project:** BOLD single-shot echo-planar images TR = 2,200 ms, TE = 30 ms, flip angle = 70°, 64 × 56 matrix, FOV = 192 × 168 mm^2^, 41 contiguous axial slices, 3.2 mm thickness, 300 volumes, acquisition time = 11 min. **Consecutive project:** BOLD single-shot echo-planar images TR = 2,500 ms, TE = 30 ms, flip angle = 70°, 64 × 64 matrix, FOV = 192 mm^2^, 44 contiguous axial slices, 3 mm thickness, 240 volumes, acquisition time = 10 min. In both projects, in addition to functional imaging, we acquired T1-weighted images which were used to increase the quality of preprocessing with the following parameters: TR = 2,300 ms, TE = 4.63 ms, flip angle = 10°, 256 × 256 matrix, FOV = 256 × 256 mm, 156 contiguous sagittal slices, 1 mm thickness.

### 2.3. Data Processing

We preprocessed raw fMRI signals using the CONN toolbox (McGovern Institute for Brain Research, MIT, USA) running under MATLAB (The Mathworks), and FSL (FMRIB Software Library v5.0, http://www.fmrib.ox.ac.uk/fslwiki, Analysis Group, FMRIB, Oxford, UK). For a detailed description of the CONN pipeline, we recommend the online documentation (https://web.conn-toolbox.org/home). Here we provide a short summary of the main steps: functional realignment and unwarping, slice-timing correction, outlier identification, direct segmentation and normalization, and functional smoothing:

(1) In the first step, realigning and unwarping procedure is used for coregistration and resampling of all scans to the first scan using b-spline interpolation (Andersson et al., 2001). (2) The temporal misalignment is corrected by time-shifting and resampling the slices using the sinc-interpolation to match the time in the middle of each acquisition (Henson et al., 1999). (3) In the outlier identification procedure, the individual volumes are flagged as potential outliers if the framewise displacement (FD) of a given volume is above the default CONN Toolbox threshold of 0.9 mm or global BOLD signal changes above five standard deviations. (Note that as another step, a lower threshold is typically applied on *mean* FD when deciding to remove a whole subject. We leave this step at the discretion of the dataset user). Averaging volumes without the potential outliers subsequently determines the new reference image. This image is further used for segmentation and normalization. (4) All samples are normalized into standard MNI space. Scans are then segmented into white matter, gray matter, and cerebrospinal fluid. For this, the structural T1 images are used to improve the quality of the registration, and the unified segmentation and normalization procedure (Ashburner and Friston, 1997) is employed. (5) Finally, fMRI signals are smoothed by convolving them with an 8 mm Gaussian kernel, increasing the signal-to-noise ratio. The preprocessed data are stored in the online repository: https://osf.io/p2kj7.

### 2.4. Functional Connectivity Estimation

**Figure 1:**
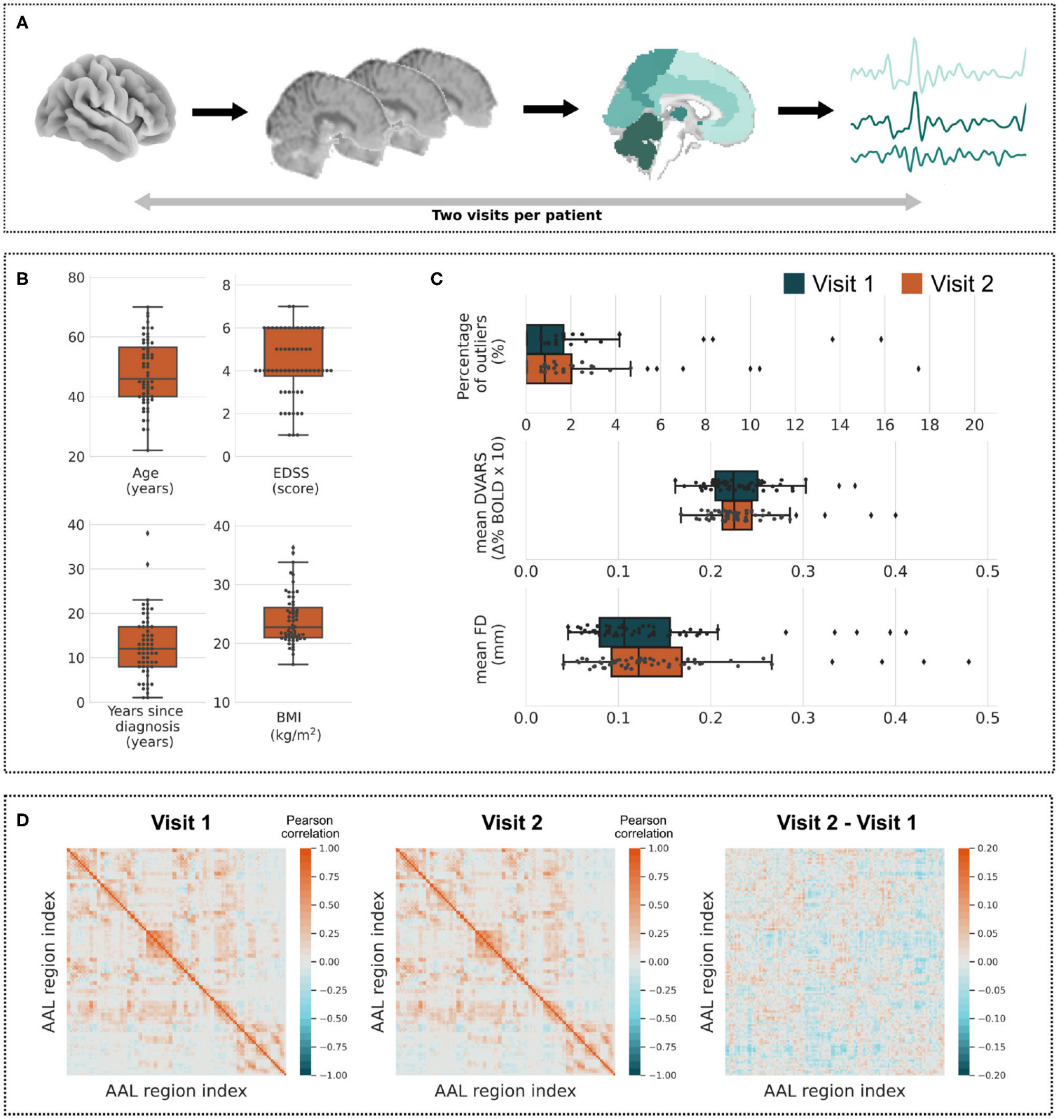
Overview of data. **(A)** Every subject's data consists of two preprocessed visits providing fMRI time series across all voxels. The time series may be averaged over atlas regions and correlated together to form functional connectivity matrices (see **D**). **(B)** Boxplots of quantitative clinical variables—Age, EDSS, Years since diagnosis, and BMI. **(C)** Distributions of the percentage of outliers, average DVARS and average FD measures across all subjects (visit 1: blue, visit 2: orange). **(D)** Average of functional connectivity matrices of the first and the second visit of all subjects and a difference of both matrices.

FC matrices were calculated by first extracting time series from 116 regions of the AAL Atlas (Tzourio-Mazoyer et al., 2002) which were linearly detrended (to remove possible signal drift) and band-pass filtered with cutoff frequencies 0.009–0.08 Hz. Filtering was performed after the regression and implemented using a discrete cosine transform windowing operation (Hallquist et al., 2013). For an intuitive visualization of these steps, see Figure 1A. The time series were then correlated, which resulted in the FC matrices. We are making the matrices available to simplify the initial data processing for users with less experience in fMRI analyses. Note that linear correlation FC matrix was proven to capture sufficiently functional connectivity (Hlinka et al., 2011) as well as its topological structure (Hartman et al., 2011) and to provide behaviorally and clinically relevant markers (Richiardi et al., 2012; Chong et al., 2019). The average FC matrices of all subjects across the first and second visit are shown in Figure 1D.

### 2.5. Outlier and Motion Artifact Detection

The elementary statistics of clinical variables are described in Table 1 with the additional information on the distribution of quantitative variables shown in Figure 1B. Two potential outliers may be identified with respect to the number of years since diagnosis—31 and 38 years. With respect to the BMI scale, there are also two potential outliers with BMI values over 35.

As described in the third step of CONN preprocessing pipeline, volumes across each subject are reviewed and, depending on the criteria, may be flagged as potential outliers. The first boxplot in Figure 1C shows the distribution of the percentage of outliers in all volumes across subjects. The median (standard deviation, minimum and maximum) percentage of flagged volumes were as follows: 0.67% (2.99, 0, 15.83%) and 0.83% (3.06, 0, 17.5%) for the first and second visit, respectively. We did identify four and six participants with a disproportionate number of flagged volumes for visits one and two, respectively.

Additionally, we estimated the two most common head motion metrics during the preprocessing: derivative of root mean square variance over voxels (DVARS) and FD (Power et al., 2014). The DVARS measure captures signal intensity changes between two consecutive volumes within a whole-brain mask (Smyser et al., 2010). FD tracks head movements between the volumes and is estimated by summing the absolute values of the differentiated realignment estimates (Power et al., 2012). By convention, the value of both measures is set to zero for the first volume. We show boxplots of the average measures across subjects in Figure 1C for better data overview. The measures can also be inspected in detail as individual time series for each subject. Apart from generally reflecting the data quality, these measures may be used as covariates in the group-level analysis.

## 3. Data Structure and Format

The structure of data is as follows: clinical.csv contains clinical descriptors of all subjects, the measures of which are in Table 1, i.e., patients' sex, age during the first visit, EDSS at the first visit, type of MS, the identifier of the pilot/consecutive project, years since diagnosis, type of therapy, and BMI. Moreover, variables “outliers_v1” and “outliers_v2” contain the absolute number of volumes flagged as outliers in visits one and two, respectively. The nifti directory contains two preprocessed fMRI acquisitions (before and after the neuroproprioceptive “facilitation and inhibition” treatment) for each subject. quality_measures contains two *.csv files DVARS.csv and FD.csv, which contain values of physical displacement measured as a function of time for each subject and session. Finally, the FC folder contains FC matrices derived from each fMRI visit together with the AAL_labels.csv file labeling regions of the AAL atlas. We also intend to release the T1 images registered to the MNI space, which were used for preprocessing. However, as they are currently a part of an ongoing student thesis project, they will be upon the study results (preprint) publication (planned within 18 months).

### 3.1. Projected Use of Data

The data may be used for studying the effect of neuroproprioceptive “facilitation and inhibition” treatment by investigating:

- changes in FC matrices
- large scale networks identified by data-driven methods such as independent component analysis
- graph-theoretical measures derived from FC matrices and others (Du et al., 2018).

Additionally, one of the visits may be used to design or validate hypotheses concerning resting state-functional processes in MS patients which are extensively studied (Bisecco et al., 2018; Bosma et al., 2018; d'Ambrosio et al., 2020).

## Data Availability Statement

The datasets presented in this study can be found in online repositories. The names of the repository/repositories and accession number(s) can be found at: https://osf.io/p2kj7/.

## Ethics Statement

The studies involving human participants were reviewed and approved by the Ethics Committee of the Faculty Hospital Královské Vinohrady. The patients/participants provided their written informed consent to participate in this study.

## Author Contributions

KŘ and JH designed the study. BB and JH coordinated the study. KŘ supervised the rehabilitation team and run the both studies. JT supervised the MRI data acquisition. JH supervised the data processing. JK preprocessed the data and revised the manuscript. BB drafted the manuscript and made the data available. All authors contributed to the manuscript revision and approved the final version for submission.

## Conflict of Interest

The authors declare that the research was conducted in the absence of any commercial or financial relationships that could be construed as a potential conflict of interest.
